# Vaccine Hesitancy Through a Global Lens: Cross-Cultural Evidence from a Special Issue

**DOI:** 10.3390/vaccines13050529

**Published:** 2025-05-16

**Authors:** Gulzar H. Shah, Tran H. Nguyen

**Affiliations:** 1 Jiann-Ping Hsu College of Public Health, Georgia Southern University, Statesboro, GA 30460, USA; 2 School of Public Health, Augusta University, Augusta, GA 30912, USA; trnguyen@augusta.edu

## 1. Introduction

Vaccine development in 1796 is among the most significant public health achievements, as it transformed global health by preventing epidemics and premature deaths, as well as eradicating life-threatening infectious diseases [1]. The World Health Organization (WHO) reports that vaccinations prevent an estimated 3.5 to 5 million premature deaths annually by preventing over 20 diseases [2]. In addition to preventing infectious diseases, some vaccines also reduce the risk of certain types of cancer by targeting infectious agents that elevate cancer risk. For instance, vaccination against Helicobacter pylori may lower the risk of stomach cancer [3], the hepatitis B vaccine significantly reduces the risk of liver cancer [4], and the human papillomavirus (HPV) vaccines are considered highly effective in preventing cervical cancer [5]. These examples underscore the significant protective benefits of vaccines beyond managing infectious diseases.

Vaccine hesitancy has been on the rise despite its proven efficacy. Such hesitancy is typically fueled by the skepticism of public health institutions, safety concerns, cultural and religious beliefs, and issues related to access and equity. In recent years, the COVID-19 pandemic has further exacerbated vaccine hesitancy, primarily due to a surge in misinformation and disruptions to routine immunization programs [6]. According to the WHO SAGE (Strategic Advisory Group of Experts), vaccine hesitancy “refers to a delay in acceptance or refusal of vaccination despite the availability of vaccination services” [7].

This Special Issue, titled “Vaccine Hesitancy”, examines the root causes of vaccine hesitancy and barriers to vaccine acceptance, emphasizing health equity and social determinants. It includes a scoping review and 16 original research studies. The studies in this Special Issue demonstrate that vaccine hesitancy is complex and multifaceted. These articles examine how demographic characteristics, social determinants, psychological factors, cultural influences, and systemic barriers affect vaccination attitudes and behaviors. While this Special Issue includes diverse contributions from multiple countries and research contexts, they collectively illuminate the multidimensional nature of vaccine hesitancy. The studies collectively highlight several essential themes, such as the impact of misinformation, confidence in scientific discourse, socio-cultural and religious convictions, health policy, and advancements in community involvement. This editorial emphasizes that the studies collectively reveal systemic impediments and prospects for targeted intervention rather than viewing each study in isolation. Readers are urged to evaluate the publications concerning their interrelated findings, which underscore the necessity of multi-sectoral and context-specific strategies to enhance global vaccine adoption.

To encourage discourse and provide a cohesive perspective from the varied contributions, it is prudent to contextualize the findings of this Special Issue within some recognized theoretical frameworks on health behavior. A comprehensive study conducted by Makadzange et al. (2023) examines the nuanced aspects of the Theory of Planned Behavior (TPB), illuminating the crucial role of perceived risk and behavioral control in shaping vaccine uptake among late adopters. In contrast, Maciuszek et al. (2023) provide indirect support for the TPB by examining the complex interplay between cognitive and social factors that influence individuals’ intentions and subsequent behaviors. Other studies highlight the significance of confidence in healthcare systems and the impact of convenience-related characteristics such as accessibility and policy transparency. This theoretical framework underscores the interrelation of the research and offers a systematic understanding of the overarching factors contributing to vaccine hesitancy, examined in this compilation of studies. This editorial discusses the principal themes listed below.

## 2. Principal Themes

### 2.1. Misinformation, Distrust, and Infodemic

Some studies on this special topic focused on individual-level misinformation, distrust in science, misconceptions, and conspiratorial thinking as detriments to vaccine development and acceptance (Tamire et al.; Maciuszek et al.). Tamire and colleagues investigated community perceptions of COVID-19 and vaccine hesitancy in selected Ethiopian cities amidst low vaccination rates that fell short of achieving herd immunity. They identified misinformation and misconceptions as the primary reasons for vaccine hesitancy. Key issues included a lack of trust in the vaccine’s safety and effectiveness and concerns about potential pain and side effects. Their findings emphasized the importance of maintaining ongoing public health campaigns and monitoring social media to counter misinformation. Steenberg et al. detailed how township residents of South Africa harbored profound mistrust of health officials and COVID-19 vaccines due to racist narratives, colonial legacies, and past injustices. Maciuszek and colleagues investigated how individuals’ trust in science, conspiratorial thinking, and religiosity negatively impacted COVID-19 vaccine uptake in Poland. This study also found that distrust in science and conspiratorial thinking were significant predictors of promoting vaccine hesitancy in others and self-vaccine hesitancy. Powell et al. examined how the synergetic effect of the epidemic of substance use disorders and the COVID-19 pandemic promoted vaccine hesitancy in the United States. Using the national sample of U.S. adults from the 2021 AmeriSpeak^®^ survey, this study assessed the relationship between negative perceptions of individuals with substance use disorders and COVID-19 vaccine hesitancy. They explored this relationship with covariates such as racial prejudice, health news sources, and other demographic factors. They showed a positive association between COVID-19 vaccine reluctance and stigma toward opioid, methamphetamine, and cocaine users. A study in China showed that after adjusting for non-modifiable indicators such as sociodemographic factors, health status, and past vaccination history, exposure to negative news about influenza vaccines and higher levels of complacency were positively correlated with parental IVH [Fan et al.]. Conversely, better knowledge about influenza vaccinations, recommendations from healthcare workers, positive attitudes toward the influenza vaccine among peers, and higher levels of confidence and convenience were negatively associated with parental IVH.

### 2.2. Social and Environmental Determinants of Vaccine Hesitancy

Lamot and Kirbiš analyzed sociodemographic and socioeconomic factors in Slovenia that had a potential influence on vaccine hesitancy. They found that higher education and improved health literacy generally reduce hesitancy. Conversely, economic deprivation increased the risk of vaccine hesitancy. Kalu and colleagues explored the connection between the social and structural determinants of health and COVID-19 vaccine hesitancy among older Americans aged 65 to 74. Utilizing data from the Health and Retirement Study, they examined the relationship between factors such as race, immigration status, internet access, Social Security income, Medicare coverage, education, and religious service attendance and vaccine hesitancy. This study concluded that economic status, higher education, and robust communicative health literacy were associated with reducing hesitancy, whereas economic hardship exacerbated it. Makadzange and colleagues studied environmental factors —such as attitudes, barriers, motivations, key influencers, and information sources—that affected vaccine uptake among individuals who adopted a “wait and see” approach, referred to as “late adopters.” Their study demonstrated that leveraging normative behavior as a social motivator for vaccination is crucial, as close social networks significantly influence vaccination decisions. Kaewkrajang et al. investigated the level of COVID-19 vaccine reluctance among Thai university students. They investigated predictors such as anxiety and optimism about the epidemic. Their findings showed that neither fear nor optimism was associated with vaccine reluctance.

### 2.3. Cultural, Religious, and Historical Contexts

Several studies cover the influence of religious, cultural, and historical factors on vaccine hesitancy. According to Steenberg et al. (2023), vaccination resistance is worsened by Africanized conspiracy beliefs and by medical pluralism, in which biomedical treatments coexist with traditional healing practices. Maciuszek et al. (2023) investigated the relationship between religious beliefs and vaccine uptake in Poland. Although religious beliefs did not directly influence vaccination rates, they were linked to less trust in science. This suggests a subtle cultural tension between religious worldviews and public health messages. These findings highlight the significance of incorporating culturally aware tactics that foster trust in public health programs to tackle vaccine hesitancy.

### 2.4. Innovations in Addressing Barriers to Vaccine Acceptance

Several articles in this issue covered novel approaches to vaccination acceptability in diverse populations. A systematic literature review by Masterson et al. (2024) found 12 studies that used tailored communication campaigns, academic–faith partnerships, and culturally meaningful outreach strategies like virtual town halls to reduce COVID-19 vaccine hesitancy in Black and African American communities. Their findings show that localized, trust-based strategies can encourage vaccine acceptability without harming vaccination efforts. Wu and Brennan-Ing found that excessive and conflicting pandemic health information reduced trust in authoritative sources for low-income older persons. The study suggested using peer-to-peer and service provider engagement in health messages to re-establish trust and vaccine confidence in vulnerable communities. A study using a repeated cross-sectional design analyzed the influence of innovative approaches by Saudi government initiatives among healthcare practitioners (Almusalami et al.). Government communication methods, policy clarity, and public education increased the rate of acceptance and advocacy among healthcare providers between the pre-vaccine approval period and the post-vaccine approval period. The findings demonstrated the government’s impact through media monitoring and collaboration with partners and stakeholders. Makadzange et al. also concluded that focused, community-centered marketing that addresses individual anxieties and leverages close social networks to enhance vaccine trust is needed.

### 2.5. Child Vaccination and Parental Attitudes

While vaccines have been proven effective in protecting children from serious illnesses, there is a growing reluctance among parents to vaccinate their children, including vaccinating them with the COVID-19 vaccine. Studies have found that parents avoided routine vaccines during the pandemic due to concerns about side effects and other obstacles (Fan et al., 2022). By analyzing these aspects, the research highlighted the importance of focused treatments that directly address parental concerns by using reliable healthcare providers as intermediaries. Szalast et al. (2025) conducted a comprehensive review of 30 studies in Poland to evaluate parental perspectives on routine childhood vaccination. They concluded that decisions to vaccinate children were driven by parental beliefs in disease prevention and vaccination safety; obstacles included a fear of adverse effects and doubt about vaccine efficacy. Abenova et al. (2024) argued that more general societal suspicion, possibly connected to the pandemic reaction, may have affected parental views on routine child immunizations.

### 2.6. Policy Influence on Vaccine Hesitancy

Studies have shown that vaccine hesitancy can be addressed through effective policy-making and enforcement. According to Shafik et al. (2023), the inconsistent adherence of clinicians and state-level implementers to U.S. Federal Government policies resulted in decreased vaccine confidence among individuals with autoimmune diseases. Low confidence was observed despite the U.S. Centers for Disease Control and Prevention (CDC) recommendations to prioritize such vulnerable individuals with autoimmune diseases during the COVID-19 vaccine rollout. Abenova et al. (2024) showed that mass COVID-19 vaccination efforts in Kazakhstan coincided with a substantial increase in childhood vaccine refusals, notably in places such as Aktobe and Atyrau. They suggested that policies leading to aggressive national-level campaigns may have unintentionally impacted public trust in routine immunizations. The authors advocated for more adaptable and transparent public health measures to address regional trust gaps and prevent long-term decreases in vaccine uptake.

### 2.7. Future Directions and Advancing Measurement

Some studies in this collection have also contributed to measurement science. Deveci et al. drew from the health behavior literature to create a scale that assesses individuals’ knowledge, attitudes, and behaviors regarding COVID-19 and evaluates the psychometric properties. Developing a thorough scale based on the Health Belief Model (HBM) and using data from more than 500 industrial zone employees in Turkey, they assessed the internal consistency, construct validity, and predictive reliability of the scale. Their studies verified that attitudes and knowledge are significant predictors of health-seeking and avoidance behaviors, essential for managing the spread of pandemics. The scale is recommended as a valuable tool for measuring COVID-19-related knowledge, attitudes, and behaviors among the workforce and the general population. Lin et al. evaluated the Motivators of COVID-19 Vaccination Acceptance Scale (MoVac-COVID19S) and developed a parent version (P-MoVac-COVID19S) to assess parents’ motivations for vaccinating their children. The P-MoVac-COVID19S, which incorporates four cognitive traits from the cognitive empowerment model, is considered a reliable and valid tool for healthcare providers to understand parents’ willingness to vaccinate their children.

This collection on vaccine hesitancy effectively addresses the call for research studies. Most articles focus on identifying system-level factors associated with vaccine hesitance. These factors include cultural beliefs about vaccines and perceptions of vaccine efficacy, effectiveness, and risks. Two articles developed and evaluated tools for assessing vaccine hesitancy among different populations. Additionally, some articles explored how governmental policies, or a lack thereof, could influence vaccine hesitancy. However, very few studies exist on effective communication strategies to increase vaccine acceptance among various populations, innovations to overcome barriers to vaccine acceptance, and assessments of vaccine hesitancy’s long- and short-term impacts. We continue to call attention to the need for more research in this area. Vaccine hesitancy has hindered efforts to improve health and prevent premature death worldwide. Additional studies on vaccine-related issues could enhance vaccine acceptance and suggest additional approaches to overcoming hesitancy.
